# Supplementary material for: Impact of Results of TTF-1 Immunostaining on Efficacy of Platinum-Doublet Chemotherapy in Japanese Patients with Nonsquamous Non-Small-Cell Lung Cancer
Source: J Clin Med. 2022 Dec 24;12(1):137. doi: 10.3390/jcm12010137 (PMC9821382; doi:10.3390/jcm12010137)
Supplement: Supplementary file 1 [file jcm-12-00137-s001.zip › jcm-2031586-supplementary.pptx]

## Slide 1
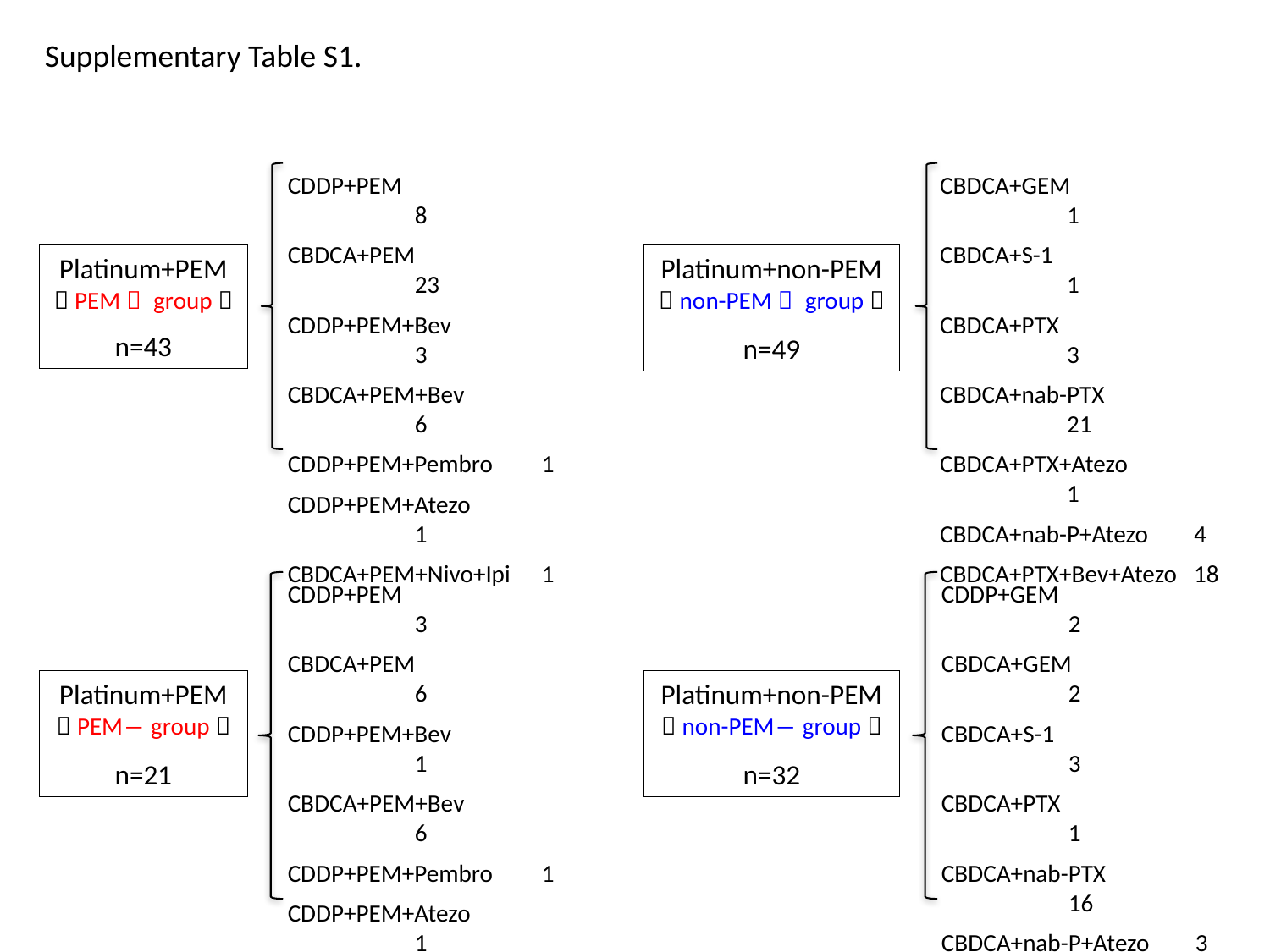

Supplementary Table S1.
CDDP+PEM			8
CBDCA+PEM		23
CDDP+PEM+Bev		3
CBDCA+PEM+Bev		6
CDDP+PEM+Pembro	1
CDDP+PEM+Atezo		1
CBDCA+PEM+Nivo+Ipi	1
CBDCA+GEM		1
CBDCA+S-1			1
CBDCA+PTX			3
CBDCA+nab-PTX		21
CBDCA+PTX+Atezo		1
CBDCA+nab-P+Atezo	4
CBDCA+PTX+Bev+Atezo	18
Platinum+PEM
（PEM＋ group）
n=43
Platinum+non-PEM
（non-PEM＋ group）
n=49
CDDP+PEM			3
CBDCA+PEM		6
CDDP+PEM+Bev		1
CBDCA+PEM+Bev		6
CDDP+PEM+Pembro	1
CDDP+PEM+Atezo		1
CBDCA+PEM+Pembro	1
CBDCA+PEM+Nivo+Ipi	2
CDDP+GEM			2
CBDCA+GEM		2
CBDCA+S-1			3
CBDCA+PTX			1
CBDCA+nab-PTX		16
CBDCA+nab-P+Atezo	3
CBDCA+PTX+Bev+Atezo	4
CBDCA+PTX+Nivo+Ipi	1
Platinum+PEM
（PEM― group）
n=21
Platinum+non-PEM
（non-PEM― group）
n=32

## Slide 2
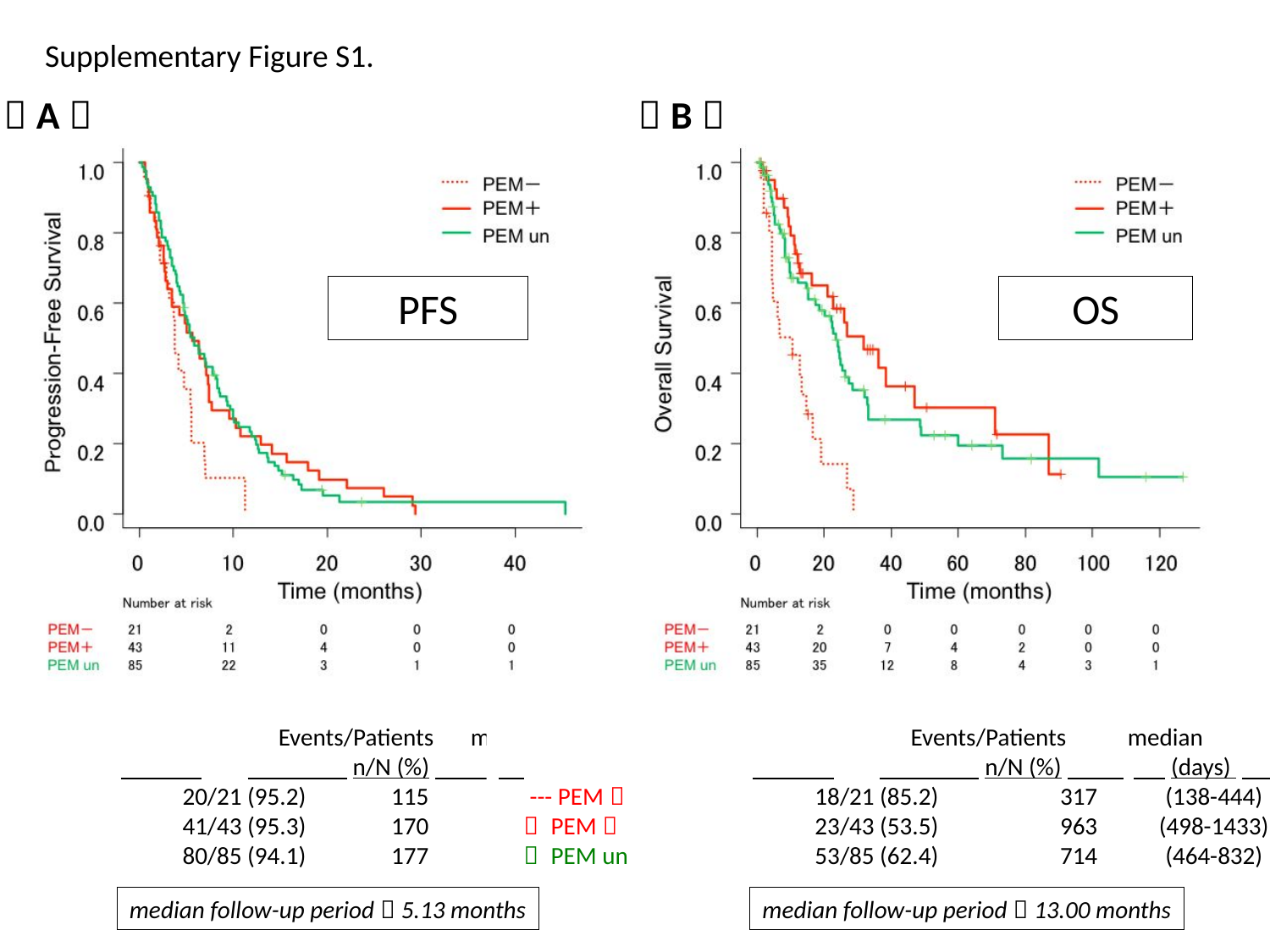

Supplementary Figure S1.
（A）
（B）
PFS
OS
			　Events/Patients　median
　		　　　 	　　　　n/N (%)　　 	 (days) 　 95%CI
　--- PEMー		　 　20/21 (95.2)	 115　　 (65-168)
　ー PEM＋		　 　41/43 (95.3)	 170　　 (91-224)
　ー PEM un 　	　　 80/85 (94.1)	 177　　(140-238)
			　Events/Patients　　median
　		　　　 	　　　　n/N (%)　　 	 　(days) 　 95%CI
　--- PEMー		　 　18/21 (85.2)	 　317　　 (138-444)
　ー PEM＋		　 　23/43 (53.5)	 　963　　(498-1433)
　ー PEM un 　	　　 53/85 (62.4)	 　714　　 (464-832)
median follow-up period；5.13 months
median follow-up period；13.00 months

## Slide 3
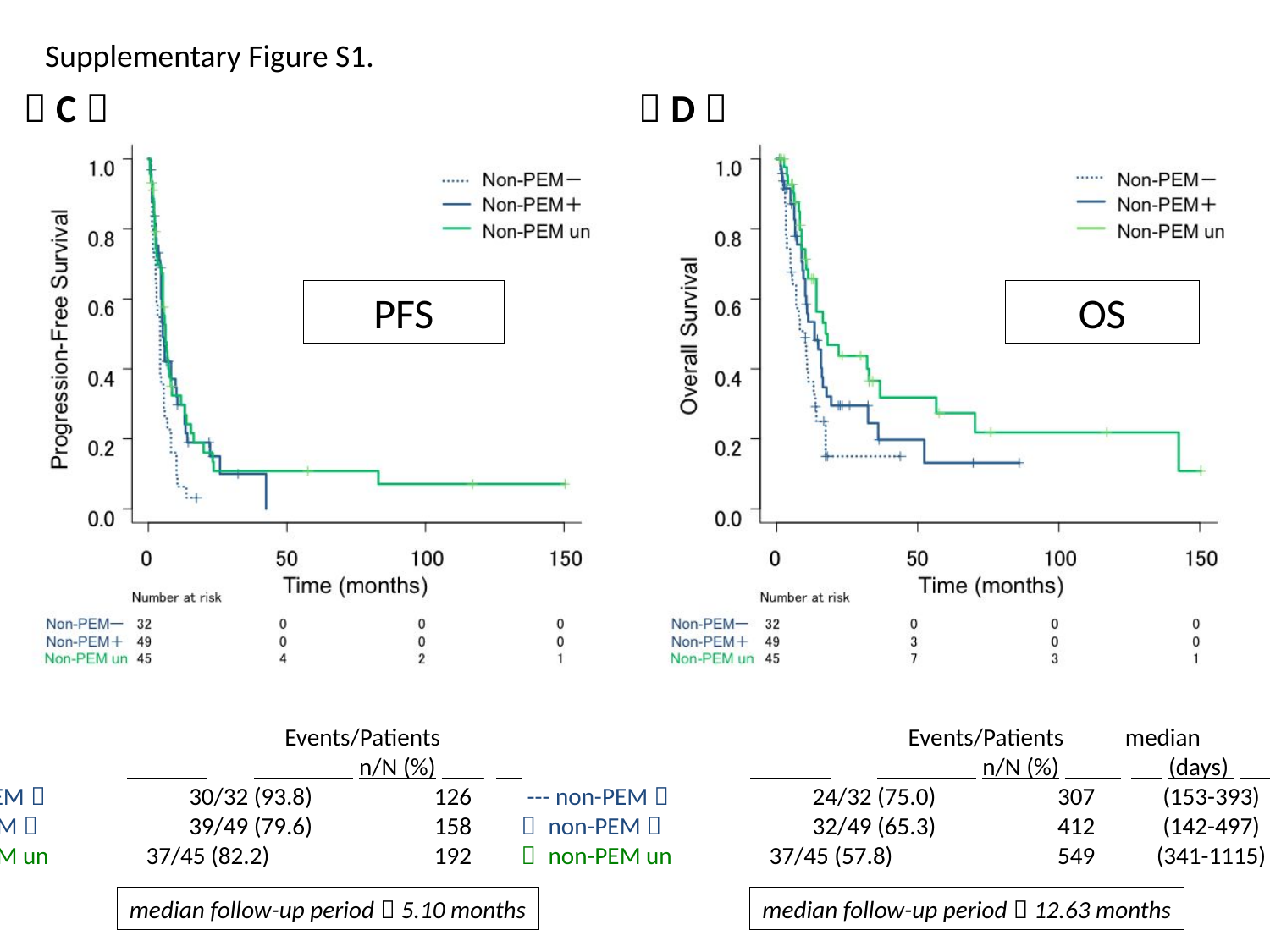

Supplementary Figure S1.
（C）
（D）
PFS
OS
			　Events/Patients　　median
　		　　　 	　　　　n/N (%)　　 	 　(days) 　 95%CI
　--- non-PEMー	　 　30/32 (93.8)	 　126　　 (77-171)
　ー non-PEM＋	　 　39/49 (79.6)	 　158　　 (142-300)
　ー non-PEM un 　　　37/45 (82.2)	 　192　　(161-252)
			　Events/Patients　　median
　		　　　 	　　　　n/N (%)　　 	 　(days) 　 95%CI
　--- non-PEMー	　 　24/32 (75.0)	 　307　　 (153-393)
　ー non-PEM＋	　 　32/49 (65.3)	 　412　　 (142-497)
　ー non-PEM un 　　　37/45 (57.8)	 　549　　(341-1115)
median follow-up period；5.10 months
median follow-up period；12.63 months

## Slide 4
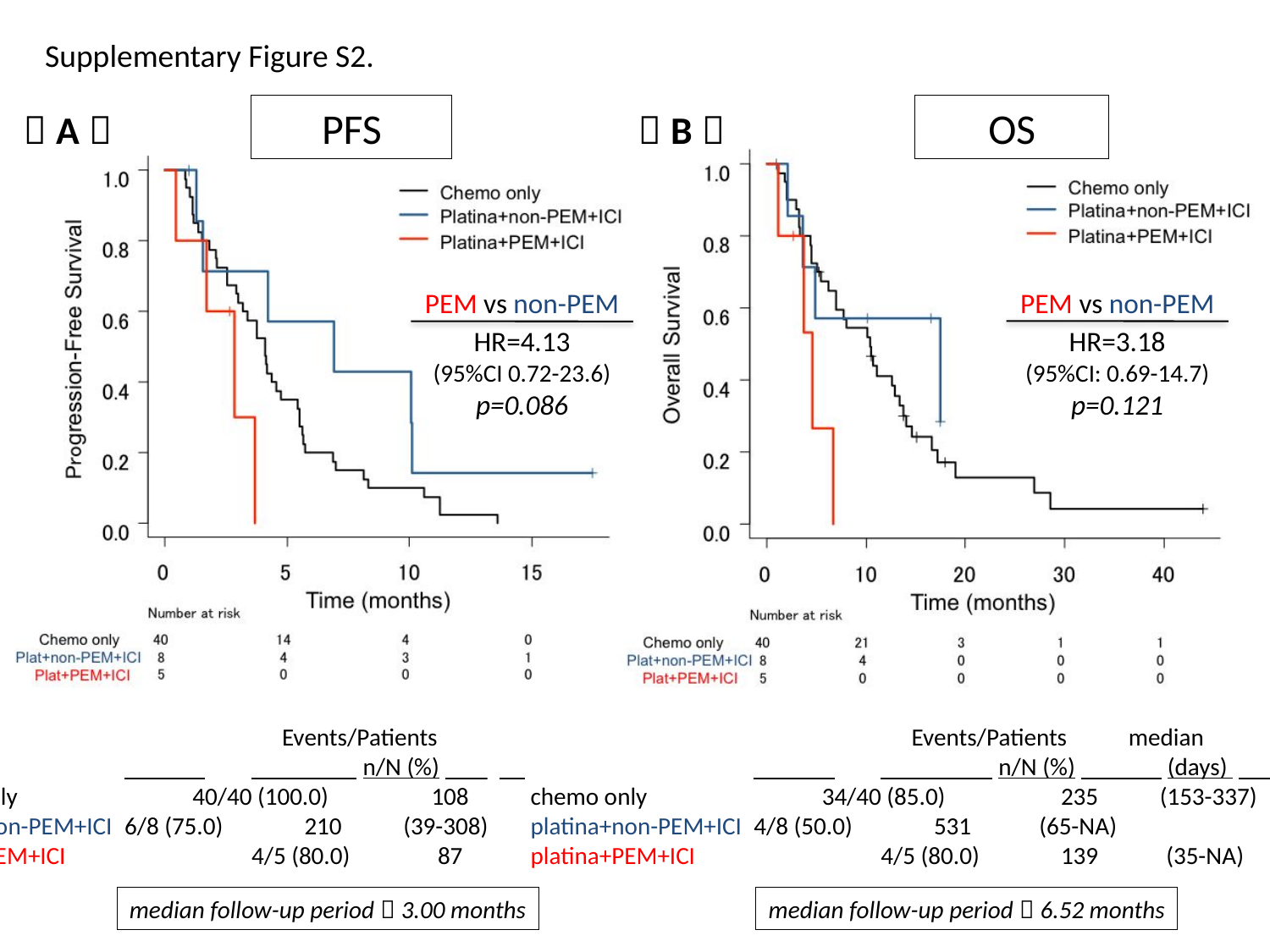

Supplementary Figure S2.
PFS
OS
（A）
（B）
PEM vs non-PEM
HR=4.13
(95%CI 0.72-23.6)
p=0.086
PEM vs non-PEM
HR=3.18
(95%CI: 0.69-14.7)
p=0.121
			　Events/Patients　　median
　		　　　 	　　　　 n/N (%)　　 	 　(days) 　 95%CI
　chemo only	　 　 40/40 (100.0)	 　108　　(64-139)
　platina+non-PEM+ICI	6/8 (75.0)	 　210　　(39-308)
　platina+PEM+ICI		4/5 (80.0)	 　 87　　 (14-NA)
			　Events/Patients　　median
　		　　　 	　　　　 n/N (%)　　 　(days) 　 95%CI
　chemo only	　 　 34/40 (85.0)	 　235　　(153-337)
　platina+non-PEM+ICI	4/8 (50.0)	 　531　　 (65-NA)
　platina+PEM+ICI		4/5 (80.0)	 　139　　 (35-NA)
median follow-up period；3.00 months
median follow-up period；6.52 months

## Slide 5
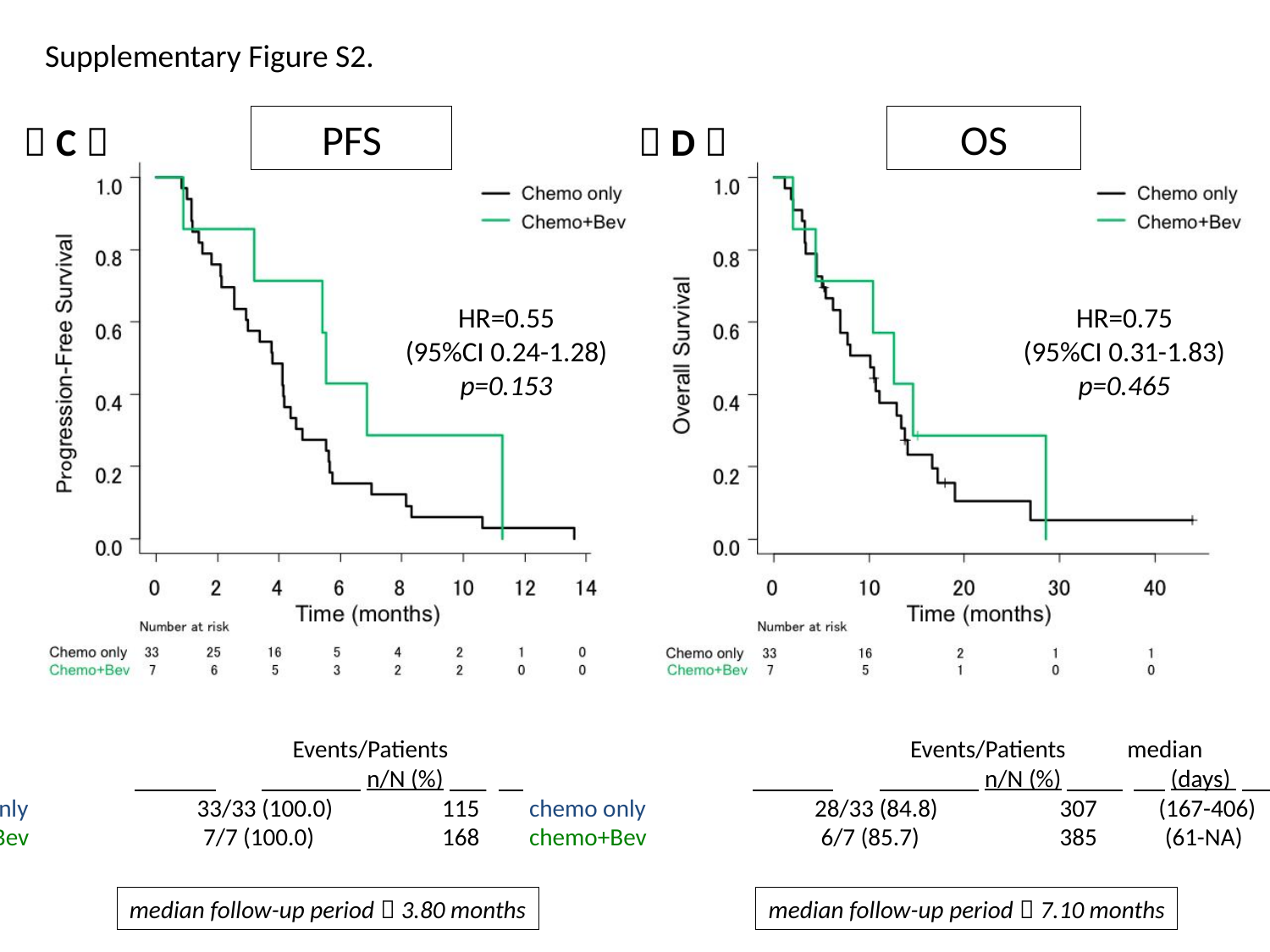

Supplementary Figure S2.
PFS
OS
（C）
（D）
HR=0.55
(95%CI 0.24-1.28)
p=0.153
HR=0.75
(95%CI 0.31-1.83)
p=0.465
			　Events/Patients　　median
　		　　　 	　　　　n/N (%)　　 	 　(days) 　 95%CI
　chemo only	　 　33/33 (100.0)	 　115　　 (77-133)
　chemo+Bev	　 　 7/7 (100.0)	 　168　　 (27-NA)
			　Events/Patients　　median
　		　　　 	　　　　n/N (%)　　 	 　(days) 　 95%CI
　chemo only	　 　28/33 (84.8)	 　307　　(167-406)
　chemo+Bev	　 　 6/7 (85.7)	 　385　　 (61-NA)
median follow-up period；3.80 months
median follow-up period；7.10 months

## Slide 6
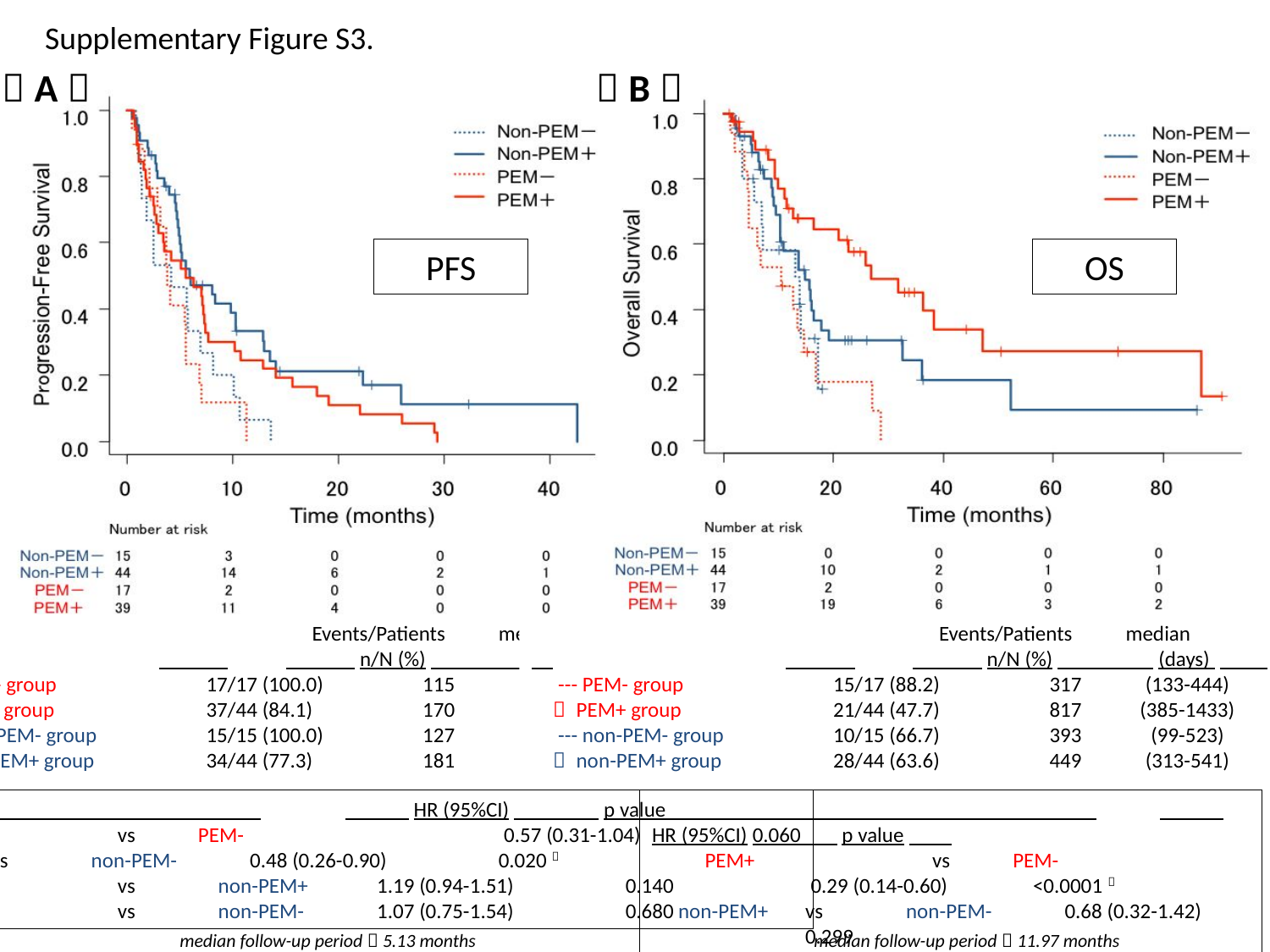

Supplementary Figure S3.
（A）
（B）
PFS
OS
			　Events/Patients　 median
　		　　　 	 　　　n/N (%)　　 　　(days) 　 　95%CI
　--- PEM- group 	　　17/17 (100.0)	 115　　 (65-168)
　ー PEM+ group	　　37/44 (84.1)	 170　　　(84-226)
　--- non-PEM- group 	　　15/15 (100.0)	 127　　 (39-210)
　ー non-PEM+ group	　　34/44 (77.3)	 181　　 (146-314)
			　Events/Patients　 median
　		　　　 	 　　　n/N (%)　　 　　(days) 　 　95%CI
　--- PEM- group 	　　15/17 (88.2)	 317　　 (133-444)
　ー PEM+ group	　　21/44 (47.7)	 817　　 (385-1433)
　--- non-PEM- group 	　　10/15 (66.7)	 393　　 (99-523)
　ー non-PEM+ group	　　28/44 (63.6)	 449　　 (313-541)
　　　　　　　　　　　　　　　　　　　　	　　　HR (95%CI)　　　　p value
　　 PEM+ 　	　vs 　　 PEM-		　 0.57 (0.31-1.04)	　0.060
　non-PEM+ 	　vs 	non-PEM-	　 0.48 (0.26-0.90) 	　0.020＊
　　 PEM+ 　 	　vs 	non-PEM+	　 1.19 (0.94-1.51) 	　0.140
　　 PEM- 　 	　vs 	non-PEM-	　 1.07 (0.75-1.54) 	　0.680
　　　　　　　　　　　　　　　　　　　　　	　　　HR (95%CI)　　　　p value
　　 PEM+ 　	　vs 　　 PEM-		　 0.29 (0.14-0.60)	<0.0001＊
　non-PEM+ 	　vs 	non-PEM-	　 0.68 (0.32-1.42) 	　0.299
　　 PEM+ 　 	　vs 	non-PEM+	　 0.74 (0.55-0.99) 	　0.041＊
　　 PEM- 　 	　vs 	non-PEM-	　 1.10 (0.72-1.66) 	　0.661
median follow-up period；5.13 months
median follow-up period；11.97 months

## Slide 7
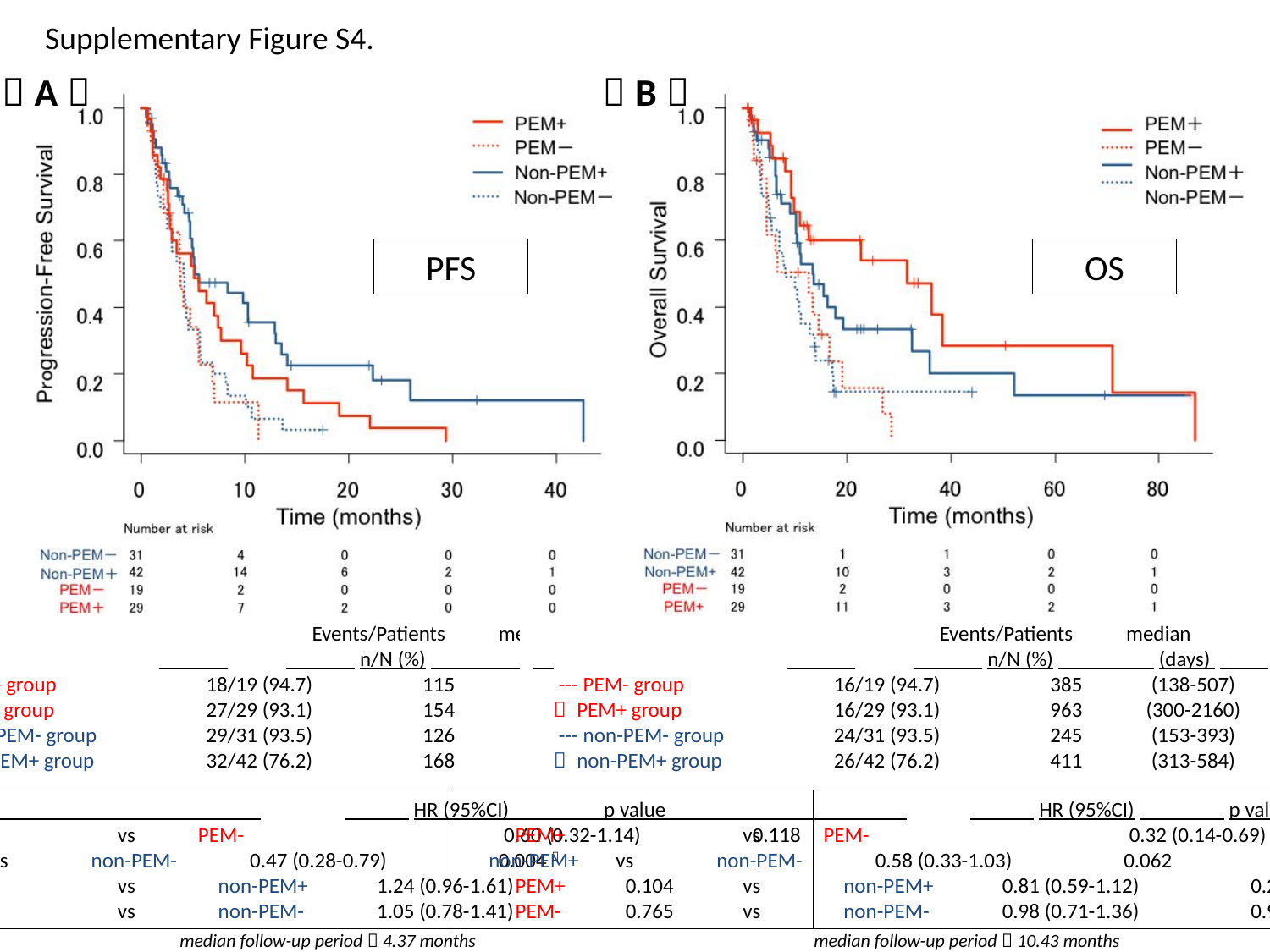

Supplementary Figure S4.
（A）
（B）
PFS
OS
			　Events/Patients　 median
　		　　　 	 　　　n/N (%)　　 　　(days) 　 　95%CI
　--- PEM- group 	　　18/19 (94.7)	 115　　 (64-165)
　ー PEM+ group	　　27/29 (93.1)	 154　　　 (82-235)
　--- non-PEM- group 	　　29/31 (93.5)	 126　　 (77-171)
　ー non-PEM+ group	　　32/42 (76.2)	 168　　 (142-393)
			　Events/Patients　 median
　		　　　 	 　　　n/N (%)　　 　　(days) 　 　95%CI
　--- PEM- group 	　　16/19 (94.7)	 385　　 (138-507)
　ー PEM+ group	　　16/29 (93.1)	 963　　 (300-2160)
　--- non-PEM- group 	　　24/31 (93.5)	 245　　 (153-393)
　ー non-PEM+ group	　　26/42 (76.2)	 411　　 (313-584)
　　　　　　　　　　　　　　　　　　　　	　　　HR (95%CI)　　　　p value
　　 PEM+ 　	　vs 　　 PEM-		　 0.60 (0.32-1.14)	　0.118
　non-PEM+ 	　vs 	non-PEM-	　 0.47 (0.28-0.79) 	　0.004＊
　　 PEM+ 　 	　vs 	non-PEM+	　 1.24 (0.96-1.61) 	　0.104
　　 PEM- 　 	　vs 	non-PEM-	　 1.05 (0.78-1.41) 	　0.765
　　　　　　　　　　　　　　　　　　　　　	　　　HR (95%CI)　　　　p value
　　 PEM+ 　	　vs 　　 PEM-		　 0.32 (0.14-0.69)	　0.004＊
　non-PEM+ 	　vs 	non-PEM-	　 0.58 (0.33-1.03) 	　0.062
　　 PEM+ 　 	　vs 	non-PEM+	　 0.81 (0.59-1.12) 	　0.214
　　 PEM- 　 	　vs 	non-PEM-	　 0.98 (0.71-1.36) 	　0.922
median follow-up period；4.37 months
median follow-up period；10.43 months
